# Reverse total shoulder arthroplasty versus locked plate fixation for proximal humeral fractures in the elderly: a systematic review

**DOI:** 10.1371/journal.pone.0317005

**Published:** 2025-02-27

**Authors:** Janette Iking, Karen Fischhuber, J. Christoph Katthagen, Sebastian Oenning, Michael J. Raschke, Josef Stolberg-Stolberg, Jeanette Köppe

**Affiliations:** 1 Institute of Biostatistics and Clinical Research, University of Muenster, Muenster, Germany; 2 Department of Trauma, Hand and Reconstructive Surgery, University Hospital Muenster, Muenster, Germany; The Affiliated Changzhou No 2 People's Hospital of Nanjing Medical University, CHINA

## Abstract

**Introduction:**

For surgical treatment of proximal humeral fractures (PHF) in older patients, there is no consensus if locked plate fixation (LPF) or reverse total shoulder arthroplasty (RTSA) yields better clinical results. The purpose of this study was to systematically review the clinical and functional outcomes of LPF and RTSA. We hypothesized that RTSA would outperform LPF in patients with PHF aged 65 years or older.

**Materials & Methods:**

A comprehensive literature search was performed on PubMed and Google Scholar from 1 July 2022 up to 12 January 2024 by two independent reviewers. Comparative studies reporting on the functional outcome using the Constant-Murley score (CMS) in patients aged 65 years or older, treated after 2012 for PHF with LPF or RTSA and with a mean follow-up time of at least 12 months were included. Ten studies with 244 LPF and 287 RTSA patients were included into the statistical analysis. We used a frequentist network meta-analysis to assess the comparative effectiveness of the treatments. Individual risk of bias of the studies was assessed using the ROB2 and ROBINS-I tools.

**Results:**

Our network meta-analysis of the CMS resulted in the following order ranked from lowest to highest: LPF, LPF +  screw augmentation, hemiarthroplasty (HA), RTSA +  cemented stem, non-surgical treatment, LPF +  fibular allograft, RTSA with an inclination angle of 135° (RTSA IA 135°), RTSA. However, none of the direct or indirect comparisons resulted in statistically noticeable differences.

**Conclusion:**

In conclusion, functional superiority of either treatment method is still unknown, with even high-powered RCT not being able to detect statistically noticeable differences in terms of function. Patient-individual factors, such as bone quality, sex and age have to be included when making treatment decisions.

## Introduction

The proximal humeral fracture (PHF) accounts for approx. 5% of all fractures, is most common in older patients and incidences are constantly rising along with demographic changes. It is the third most frequent osteoporotic fracture and about two-thirds of the patients have female sex [1,2]. About 46% of PHF are treated surgically due to fracture complexity or patient-specific factors [3]. Amongst the most frequently performed surgical procedures are open reduction and internal fixation using locked plate fixation (LPF) and reverse total shoulder arthroplasty (RTSA) [4]. While joint preserving surgery has traditionally been the choice of treatment, total joint replacement by RTSA is becoming increasingly popular, especially in older patients [5,6]. However, there is no consensus whether LPF or RTSA should be performed [7].

To date, LPF remains the most commonly performed surgical procedure, but it continues to be a challenge for surgeons as high complication rates have been reported [5,8]. Frequent complications include intraarticular screw penetration, avascular necrosis, varus malunion, subacromial impingement, nonunion and infection [9,10]. Hence, 10-14% of patients need revision surgery after LPF, amongst older patients even up to 29% [11,12]. RTSA is an alternative treatment option and should be considered in cases of varus displaced four-part fractures, multiple-part fractures with a small humeral head fragment, non-reducible head-split fractures, depressed fractures with more than 40% joint involvement and rotator cuff insufficiency [13]. Although recent studies also suggested high complication rates for RTSA, they also reported fewer revision surgeries after primary RTSA compared to LPF [5,14]. Furthermore, evidence is increasing that RTSA might outperform LPF clinically [14–16]. In addition, it has been shown that primary RTSA causes fewer complications than salvage RTSA after failed LPF with controversial data on functionality [10,13,17–19]. To sum up, there is still no higher level evidence for superiority in clinical outcome or functionality of LPF or RTSA in the surgical treatment of PHFs in older patients.

Hence, the purpose of this meta-analysis was to report the clinical outcomes given as Constant-Murley scores (CMS) after primary RTSA or LPF in patients aged 65 years or older, including different treatment variants to improve the power of the network analysis. We hypothesized that RTSA yields better functional results than LPF.

## Methods

### Literature search and selection criteria

The review was prepared according to the Preferred Reporting Items for Systematic Reviews and Meta-Analysis (PRISMA) guidelines without a registration protocol (see supplementary file S1 and S2). A comprehensive search of the literature was performed by two independent reviewers in the bibliographic databases PubMed and Google Scholar being up to date on 12 January 2024. The following research terms were used: “proximal humer*” AND fracture AND (arthroplasty OR plate OR fixation) with the following filters applied (when applicable): Language: English, German; Article type: clinical study, clinical trial, comparative study, controlled clinical trial, multicentre study, observational study, randomized controlled trial; Publication date: 2012 and onwards. Comparative studies including RCT describing functional outcomes using the CMS after LPF or RTSA to treat PHF were included. Only articles describing patients aged 65 years or older treated after 2011 were considered. Studies with a follow-up time of less than 12 months and more than 60 months were also excluded. Articles were screened by title and abstract and duplicates were removed. Articles that initially met the criteria were then further evaluated by a full-text review. Reasons for exclusion of all studies left after abstract screening can be found in Table S3.

### Risk-of-bias assessment

RCT and non-randomized trials were included in the analysis. Hence, the risk of bias was assessed by two independent reviewers using two different types of analysis tools, the ROB 2 tool [20] for assessing RCTs and the ROBINS-I tool [21] for assessing non-randomized trials, as described previously [22].

### Statistical analysis

The standardized mean difference of the CMS was used as the effect size of the examined network meta-analysis. The data extraction was complete in terms of effect size. In some cases, standard deviations (SD) had to be computed based on reported confidence intervals or standard errors, but there was no need for an imputation. In two cases (**Table 1**, cases are marked with “*Δ*”) only a range of the mean follow-up time was given. A rigorous approximation of the SD was computed by assuming the range to be the 95% confidence interval. Those SD were not included in the statistical analysis but were solely calculated to compare the dispersion of the studies. In one study, the results of the intention-to-treat (ITT) as well as of the per-protocol (PP) population were reported [31]. In the main analysis, we considered the results of the ITT population and performed a sensitivity analysis for the PP population. To assess the comparative effectiveness, a frequentist network-analysis based on a random effects model was used. Heterogeneity of the model was measured with the Q-statistic. Variables describing the study population, such as gender, age and fracture type, were presented descriptively in **Table 1**, if information was found. Since a known CMS was defined as an inclusion criterion, no missing data occurred in the study. All calculations were carried out with R Studio Version 1.3 [32] and R Version 4.1.2 [23] using the netmeta package [33].

**Fig 1:**
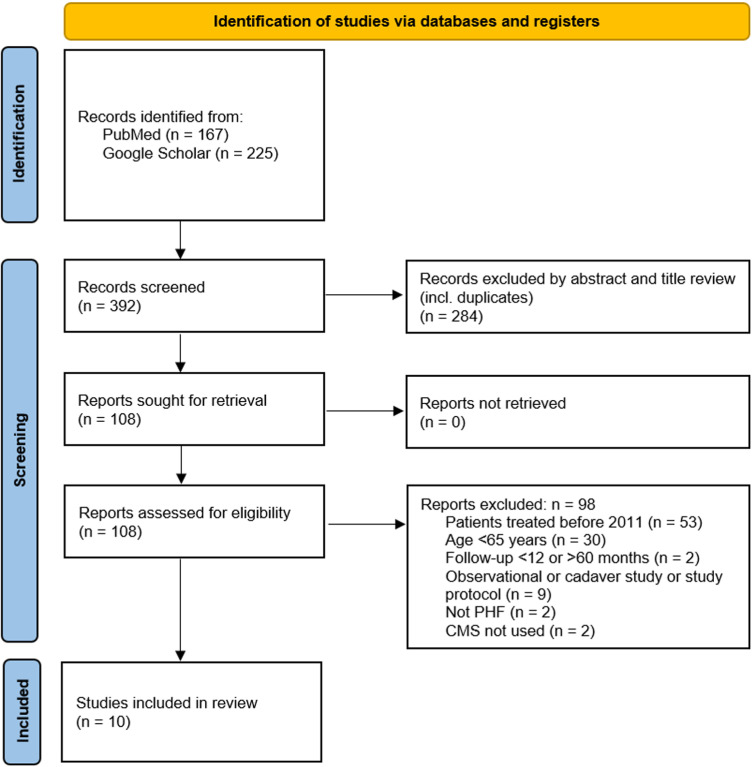
Flow diagram of search strategy and study selection.

**Table 1:** Overview of the included studies and baseline characteristics. CMS, Constant-Murley score. FU, follow-up. HA, hemiarthroplasty. HSF, Head-splitting fracture. LPF, locked plate fixation. NS, not specified. RTSA, reverse total shoulder arthroplasty. *Δ* approximation by using range as 95%CI.

Study	Study design	Treatment	Sample size (baseline)	Age in years mean (±SD)	Female N (%)	Fracture type	Sample size at FU	Follow-up in months mean (±SD)	CMS mean ( ± SD)	Data extractor and date
Fraser et al. 2020 [16]	RCT	LPF RTSA	60 64	74.7 (±6.5) 75.7 (±6.1)	52 (87%) 59 (92%)	2-part (48%) 3-part (52%) 2-part (41%) 3-part (59%)	49 57	12 (±0) 12 (±0)	54.3 ( ± 20.8) 62.8 ( ± 20.8)	JI/KF, 4.12.2023, met all inclusion criteria
Hengg et al. 2019 [23]	RCT	LPF LPF + screw augmentation	34 33	76.1 (±6.2) 77.5 ( ± 7.4)	29 (85%) 26 (79%)		23 27	12 (±0) 12 (±0)	66.6 ( ± 18.4) 64.4 ( ± 19.1)	JI/KF, 4.12.2023, met all inclusion criteria
Holschen et al. 2022 [24]	Non-randomized	RTSA (IA 155°) RTSA IA 135°	30 28	78.0 (NS) 79.0 (NS)	27 (90%) 26 (93%)	2-part (37%) 3-part (63%) 2-part (39%) 3-part (61%)	30 28	35 ($\pm 8.3^{△})$^Δ^) 30 ($\pm 5.4^{△})$^Δ^)	63.1 ( ± 16.8) 60.5 ( ± 12.8)	JI/KF, 4.12.2023, met all inclusion criteria
Jonsson et al. 2021 [25]	RCT	RTSA HA	41 43	80.4 (±4.5) 78.6 (±4.8)	39 (95%) 37 (86%)	3-part (49%) 4-part (46%) 3-part (47%) 4-part (37%)	35 32	12 (±0) 12 (±0)	54.8 ( ± 16.2) 45.7 ( ± 19.1)	JI/KF, 4.12.2023, met all inclusion criteria
Jorge-Mora et al. 2019 [26]	Non-randomized	RTSA cemented RTSA non-cemented	24 34	77.6 (NS) 76.5 (NS)	24 (100%) 31 (91%)	NS (3- and 4-part)	24 34	26 ($\pm 12.48^{△}$^Δ^)	53.0 ( ± 12.0) 60.0 ( ± 12.0)	JI/KF, 4.12.2023, met all inclusion criteria
Lopiz et al. 2019 [27]	RCT	RTSA Non-OP	29 30	82.0 (±3.2) 85.0 (±4.8)	25 (86%) 26 (87%)	3-part (13%) 4-part (87%) 3-part (17%) 4-part (83%)	29 30	12 (±0) 12 (±0)	61.7 ( ± 12.1) 55.7 ( ± 12.4)	JI/KF, 4.12.2023, met all inclusion criteria
Siebenbürger et al. 2019 [28]	Non-randomized	LPF LPF + screw augmentation	55 39	76.6 (±11.1) 78.2 (±10.2)	43 (78%) 32 (82%)	2-part (36%) 3-part (40%) 4-part (24%) 2-part (41%) 3-part (38%) 4-part (21%)	55 39	24 (±0) 24 (±0)	62.6 ( ± 17.4) 63.7 ( ± 18.5)	JI/KF, 4.12.2023, met all inclusion criteria
Zhao et al. 2019 [29]	Non-randomized	LPF LPF + fibular allograft	21 21	69 (±7.2) 68.8 (±6.3)	9 (43%) 12 (57%)	3-part (67%) 4-part (33%) 3-part (71%) 4-part (29%)	21 21	12 (NS) 12 (NS)	79.7 ( ± 9.14) 86.0 ( ± 7.6)	JI/KF, 4.12.2023, met all inclusion criteria
Klug et al. 2020 [16]	Non-randomized	LPF RTSA	30 30	72.5 ( ± 6.3) 76 ( ± 6.7)	25 (83%) 25(83%)	3-part (33%) 4-part (60%) HSF (7%) 3-part (3%) 4-part (57%) HSF (40%)	30 30	49 ($\pm 9.75^{△}$^Δ^) 38 ($\pm 7.13^{△}$^Δ^)	81.4 ( ± 17.2) 69.9 (±26)	JI/KF, 4.12.2023, met all inclusion criteria
Lanzetti et al. 2022 [30]	Non-randomized	LPF RTSA	66 72	73 ( ± 2.9) 76 ( ± 2.9)	29 (44%) 40 (68%)	3-part (36%) 4-part (64%) 3-part (38%) 4-part (63%)	66 72	53 ($\pm 11.94^{△}$^Δ^)	53 ( ± 5) 85 (±7)	JI/KF, 4.12.2023, met all inclusion criteria
Overall			**784**	**76.5**	**616 (79%)**		**732**	**26.59**		

## Results

We initially identified 392 references of interest. Of that, 108 articles were further retrieved for full-text review. Finally, 10 studies with 244 LPF and 287 RTSA patients met our inclusion criteria as outlined in **Fig 1**. The included studies featured three direct comparisons between LPF and RTSA, two comparisons between LPF and LPF +  screw augmentation, and one comparison each between LPF and LPF +  fibular allograft, RTSA and hemiarthroplasty (HA), non-operative treatment and RTSA, cemented RTSA and non-cemented RTSA with locked stems, and RTSA with an inclination angle (IA) of 155° without a lateral offset and RTSA with an IA of 135° and a lateral offset of 4 mm. Non-cemented RTSA and RTSA with an IA of 155° without a lateral offset were considered as standard procedure and therefore included in the RTSA group. A total of 784 patients were included (79% female sex, mean age 76.5 years) with a mean follow-up of 26.6 months. **Table 1** gives a full overview of the included studies.

**Fig 2:**
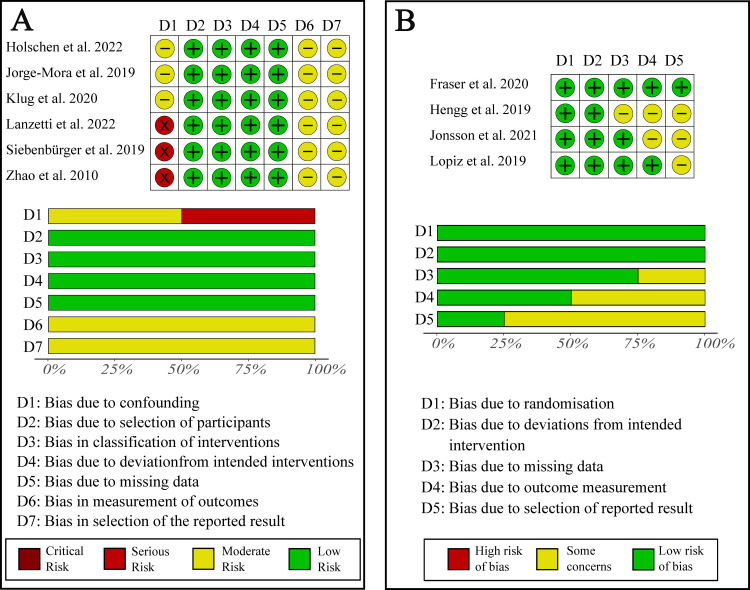
Overview of risk of bias assessment for the included studies. **(A)** ROBINS-I risk of bias assessment for included non-randomized trials. **(B)** ROB 2 risk of bias assessment for included randomized controlled trials.

The risk of bias analysis concluded that all six included non-randomized controlled trials had a low risk for selection bias, bias in classification of interventions, bias due to deviation from intended interventions and bias due to missing data. However, they all showed some moderate risk for a bias in measurement of outcomes and bias in selection of the reported result. Furthermore, three studies had a moderate and three studies had a high risk for bias due to confounding (**Fig 2A**). The four included randomized controlled trials all had a low risk for bias due to randomisation and bias due to deviation from intended interventions. One study raised some concerns for bias due to missing data, two studies for bias due to outcome measurement and three studies for bias due to selection of the reported results (**Fig 2B**).

**Fig 3:**
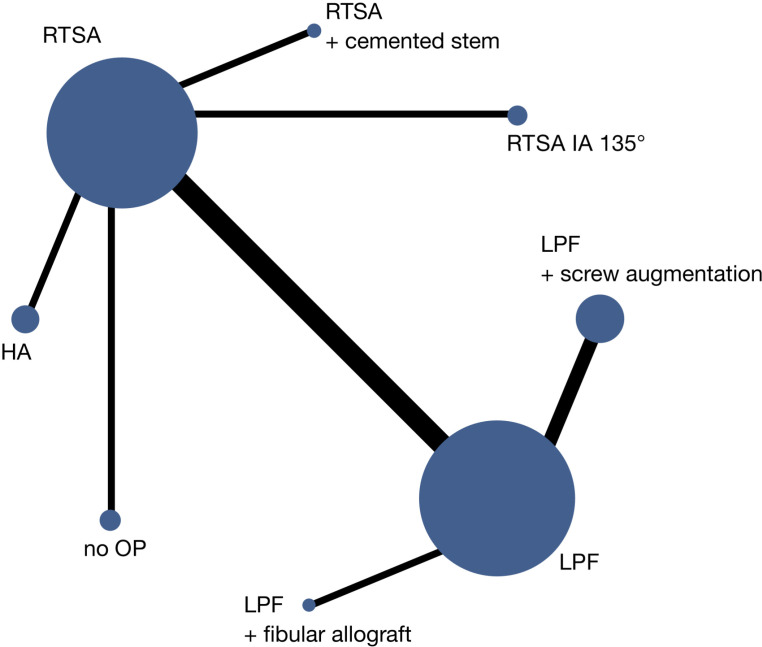
Connectivity diagram of the analysed treatments. The size of the circles represents the sample size of the treatment groups.

Ten studies and eight treatment varieties (LPF, LPF +  screw augmentation, LPF +  cemented screw allograft, RTSA, RTSA IA 135°, RTSA +  cemented stem, HA, non-operative) were included in the statistical analysis (**Table 2**). Two treatment arms were compared in all studies and thus no correction for multiple arms was required. There were three occurrences of the design LPF vs. RTSA and two occurrences of the design LPF vs. LPF +  screw augmentation. Other designs only occurred once (**Fig 3**). Within-design heterogeneity was measured by the Q-Test (Q = 84.58, p < 0.001). The between-design heterogeneity was quantified by $\tau^{2}$= 375.22 ($I^{2}$=96.5% [95%CI: 93.5%-98.1%]) and thus a network meta-analysis with random effects was used. For a complete overview of the results of the network meta-analysis, see **Table 2**. Due to the high heterogeneity within the designs and thus the use of random effects within our design, no statistically noticeable effect could be detected.

**Table 2:** Pairwise difference of the mean CMS resulting from the network meta-analysis with a random effects model. HA, hemiarthroplasty. IA, inclination angle. LPF, locked plate fixation. RTSA, reverse total shoulder arthroplasty.

	Treatment 1	Treatment 2	#Studies	Mean difference CMS (T2-T1)	95%-CI	P-value
Direct estimates	HA	RTSA	1	-9.1	[-48.0, 29.8]	**0.647**
	LPF	LPF + screw augm.	2	0.5	[-27.1, 28.1]	**0.971**
	LPF	LPF + fibular allograft	1	-6.3	[-44.6, 32.0]	**0.748**
	LPF	RTSA	3	-10.3	[-32.6, 12.1]	**0.370**
	non-operative	RTSA	1	-6.0	[-44.5, 32.5]	**0.760**
	RTSA	RTSA + cemented stem	1	7.0	[-31.5, 45.5]	**0.721**
	RTSA	RTSA IA 135°	1	2.6	[-36.1, 41.3]	**0.895**
Indirect estimates	HA	LPF		1.2	[-43.7, 46.0]	**0.960**
	HA	LPF + screw augm.		1.7	[-51.0, 54.4]	**0.951**
	HA	LPF + fibular allograft		-5.1	[-64.1, 53.9]	**0.865**
	HA	non-operative		-3.1	[-57.8, 51.6]	**0.912**
	HA	RTSA + cemented stem		-2.1	[-56.8, 52.6]	**0.940**
	HA	RTSA IA 135°		-6.5	[-61.4, 48.4]	**0.817**
	LPF	non-operative		-4.3	[-48.8, 40.3]	**0.852**
	LPF	RTSA + cemented stem		-3.3	[-47.8, 41.3]	**0.971**
	LPF	RTSA IA 135°		-7.7	[-52.4, 37.1]	**0.738**
	LPF + screw augm.	LPF + fibular allograft		-6.8	[-54.0, 40.4]	**0.778**
	LPF + screw augm.	non-operative		-4.8	[-57.1, 47.6]	**0.858**
	LPF + screw augm.	RTSA		-10.8	[-46.3, 24.8]	**0.553**
	LPF + screw augm.	RTSA + cemented stem		-3.8	[-56.1, 48.6]	**0.888**
	LPF + screw augm.	RTSA IA 135°		-8.2	[-60.7, 44.4]	**0.761**
	LPF + fibular allograft	non-operative		-4.8	[-56.7, 60.8]	**0.946**
	LPF + fibular allograft	RTSA		-4.0	[-48.3, 40.4]	**0.861**
	LPF + fibular allograft	RTSA + cemented stem		3.0	[-55.7, 61.8]	**0.919**
	LPF + fibular allograft	RTSA IA 135°		-1.4	[-60.3, 57.5]	**0.964**
	non-operative	RTSA + cemented stem		1.0	[-53.4, 55.4]	**0.971**
	non-operative	RTSA IA 135°		-3.4	[-58.0, 51.2]	**0.903**
	RTSA + cemented stem	RTSA IA 135°		-4.4	[-59.0, 50.2]	**0.875**

**Fig 4:**
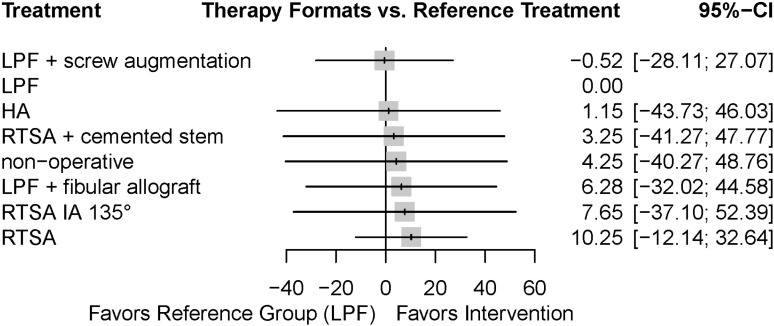
Forest plot of the analysed treatments. The mean difference of the CMS compared to the reference group (LPF) is shown as a grey square with the corresponding 95%CI. Note that a higher CMS results in a positive difference, i.e., the intervention is superior to the reference treatment. However, there was no statistically noticeable difference between the interventions. HA, hemiarthroplasty. IA, inclination angle. LPF, locked plate fixation. RTSA, reverse total shoulder arthroplasty.

The comparison of the treatment groups is visualized in a forest plot with LPF as a reference treatment (**Fig 4**). The treatments were ranked with respect to the achieved P-scores, which measures the mean extent of certainty that a treatment is better than all competing treatments. RTSA was ranked first (0.657), followed by RTSA IA 135° (0.560), LPF +  fibular allograft (0.541), non-operative treatment (0.501), RTSA +  cemented stem (0.484), HA (0.448), LPF +  screw augmentation (0.411) and LPF (0.399). Results for the designs including LPF +  screw augmentation changed, when considering the per-protocol population (PP) instead of the intention-to-treat population; however, this did not affect the heterogeneity of the overall model nor did it change the direction of the effects or the statistical conclusion. The results of the other designs remained unchanged.

**Fig 5:**
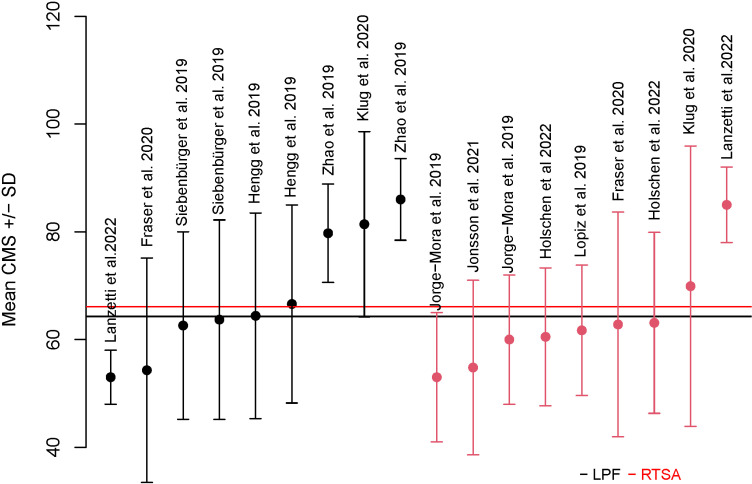
Reported mean CMS ±SD for each included study. The mean CMS for all LPF variants was 64.3 ( ±  19.0), shown as a black line. The mean CMS for all RTSA variants was 66.1 ( ±  19.0), shown as a red line. CMS, Constant-Murley score. LPF, locked plate fixation. RTSA, reverse total shoulder arthroplasty.

The calculated overall absolute mean CMS for all RTSA variants was 66.1 ( ± 19.0) and 64.3 ( ± 19.0) for all LPF variants. The average reported CMS for LPF and RTSA for each study is visualized in **Fig 5**.

## Discussion

The best surgical treatment for PHFs in older patients remains highly controversial. Although some randomized controlled trials (RCT) and comparative studies have been published on this topic in recent years, the sample sizes were small and groups were difficult to compare.

Within this study, a network meta-analysis of the functional outcome by means of CMS on the two most commonly performed procedures, LPF and RTSA, was performed. Since surgical techniques as well as implant designs have evolved over time, only studies with patients treated after 2011 were included. Although RTSA ranked highest according the P-score, there was no statistically noticeable difference between LPF and RTSA regarding the functional outcome by means of the CMS, which is consistent to the heterogeneous results previously reported by other authors (**Fig 4**) [13–16,34–36]. Similarly, direct and indirect comparisons of the eight analysed treatment variants yielded no significant differences (**Table 2**).

Several previously published studies comparing RTSA with LPF reported favourable outcomes for RTSA [15,34–37]. Direct comparison between RTSA and LPF was conducted by Fraser *et al.* showing a mean CMS of 68 and 54.6 points (p <  0.001) two years after surgery as well as in a systematic review by Heo *et al.* including four additional retrospective studies with a total CMS of 73 and 79 points (p =  0.99), respectively [15,16,37–39]. A recent study by Zhou *et al.* suggested a minimal clinically important difference for CMS after RTSA of 7.2, 6.6 and 9.3 points after 3, 6 and 12 months, respectively [40]. Looking at the achieved range of motion (ROM) after implementation of RTSA and LPF, it has been shown that RTSA significantly improved forward flexion, was comparable in abduction, but inferior in external rotation compared to LPF [13,14]. A systematic review from 2023, however, also reported a significantly better external rotation for RTSA compared to LPF [41] and other studies even reported a superiority of LPF in ROM in all directions [16]. This shows once more, how inconclusive the data on this topic currently are. One reason for this might be that clinical outcome and ROM highly depend on the quality of fracture reduction after LPF [42].

Re-operation rates, complications, major adverse events, mortality, pain, and quality of life should also be considered, in addition to functional outcome measures. Data on complication and revision rates vary widely for both surgical treatments. LPF was associated with frequent complication rates of up to 49% and high re-operation rates of up to 29% in older patients [11,12,43–48]. The reported complication rates for RTSA vary widely, ranging from 0% to up to 68% in older studies [13]. Recent studies suggested increased complication rates after RTSA in comparison to LPF (increase by 42%), but fewer revision surgeries (decrease by 63%) [5,14]. Hence, a learning curve can be observed for RTSA, as older studies report higher complication rates than recent studies [13]. A recently published systematic review comparing surgical approaches after PHF in adults even found that LPF and RTSA are similar, not only regarding clinical outcomes, but also regarding complication rates, highlighting again that different studies can produce vastly different results [41]. An individualized approach on treating PHF might overcome these inconclusive results on clinical and functional outcomes after surgical (or non-surgical) treatment of PHF [49]. A study analysing German health insurance data of 8 years including more than 50.000 patients was able to show that after adjustment to the patients risk profile, RTSA resulted in significant lower mortality and fewer major adverse events in the long-term [50]. However, the in-hospital rates for perioperative complications and major adverse events for RTSA were increased compared to LPF, even after adjusting for factors such as age, sex, and risk profile [5]. Moreover, it was observed that male sex was associated with an increased risk in mortality and complications after surgical treatment of PHF [51]. Hence, in an approach to individualize surgical decision making, patients need to be informed according to their risk profile. Only the combined evaluation of expected complications and functionality will ultimately result in better outcomes for both procedures, LPF and RTSA.

This systematic review has several limitations: (1) There was only one comparison for each treatment modality apart from RTSA vs. LPF (n = 3) and LPF vs. LPF +  screw augmentation (n = 2), which limits the significance of our conclusion. (2) The different percentages of two-, three-, and four-part fractures in different studies, the varying and relatively short times of follow-up, the variability of technical skills of the operating surgeons in different studies as well as different surgical approaches, the subjective component of the CMS, and the variability in post-operative care may influence the functional outcome. (3) We only included a small number of studies with CMS as an outcome indicator; however, other factors such as re-operation rates, complications, major adverse events, mortality, pain, and quality of life should also be considered. In addition, many of the included studies either presented with low level of evidence and/or high risk of bias in some categories. Therefore, the results should be interpreted with caution.

Concluding, within the present meta-analysis, there is no difference between LPF and RTSA regarding the functional outcome by means of the CMS. Although RTSA achieved the highest average point values, no statistically noticeable difference was observed. Several recent studies have been published to find a definitive answer to the question on the optimal surgical technique for older patients with PHF, however, the reported data were either not statistically significant or yielded contradicting results. Possibly, the correct answer to the question at hand can be found in an individual approach, assessing the risk for each patient including age, sex, fragility, bone quality and fracture classification.

## Supporting information

S1 File PRISMA checklist. (PDF)

S2 File PRISMA abstract checklist. (PDF)

S3 File Table of all studies with reasons for exclusion. (XLSX)
