# Correction: Reverse total shoulder arthroplasty versus locked plate fixation for proximal humeral fractures in the elderly: a systematic review

**DOI:** 10.1371/journal.pone.0322033

**Published:** 2025-04-04

**Authors:** Janette Iking, Karen Fischhuber, J. Christoph Katthagen, Sebastian Oenning, Michael J. Raschke, Josef Stolberg-Stolberg, Jeanette Köppe

There are errors in the author affiliations. The correct affiliations are as follows:

Janette Iking^1^, Karen Fischhuber^2^, J. Christoph Katthagen^1^, Sebastian Oenning^1^, Michael J. Raschke^1^, Josef Stolberg-Stolberg^1^, Jeanette Köppe^2^

**1** Department of Trauma, Hand and Reconstructive Surgery, University Hospital Muenster, Muenster, Germany, **2** Institute of Biostatistics and Clinical Research, University of Muenster, Muenster, Germany.
